# A Screening Assay for Bile Acid-Transforming Microorganisms Using Engineered Bacterial Biosensors

**DOI:** 10.3390/bios15110716

**Published:** 2025-10-29

**Authors:** Debora Dallera, Daniele Pastorelli, Massimo Bellato, Angelica Frusteri Chiacchiera, Francesca Usai, Maria Gabriella Cusella De Angelis, Paola Brun, Paolo Magni, Lorenzo Pasotti

**Affiliations:** 1 Department of Electrical, Computer and Biomedical Engineering, University of Pavia, Via Ferrata 5, 27100 Pavia, Italy; 2 Centre for Health Technologies, University of Pavia, Via Ferrata 5, 27100 Pavia, Italy; 3 Department of Molecular Medicine, University of Padua, Via Gabelli 63, 35121 Padua, Italy; 4 Department of Information Engineering, University of Padua, Via Gradenigo 6b, 35131 Padua, Italy; 5 Human Anatomy Unit, Department of Public Health, Experimental and Forensic Medicine, University of Pavia, Via Forlanini 8, 27100 Pavia, Italy

**Keywords:** bile salts, bacterial sensor, *Escherichia coli*, lactic acid bacteria, fluorimetric assay, specificity, synthetic biology, TcpP, TcpH, calibration curve

## Abstract

Bile salt hydrolase (BSH) enables microbial-mediated deconjugation of bile acids (BAs) in the gastrointestinal tract. BSH enzymes initiate bile acid metabolism by catalyzing the first, essential deconjugation step. Due to the strict connection between dysregulations of the BA pool and human or animal diseases, identification and characterization of strains with BSH activity are relevant for both healthcare and agroindustry. However, current methods are expensive, poorly sensitive, or require complex procedures. Here, a BSH screening assay for cultivated microbes is proposed, based on a bacterial biosensor that reports the concentration of different BA types via fluorescence. Although the biosensor is broadly responsive to various bile acids, the assay was designed to guarantee specificity by testing individual primary BAs within controlled concentration ranges. The assay was evaluated on two recombinant *Escherichia coli* strains bearing BSH genes from *Lactobacillus johnsonii* PF01 and a BSH-positive probiotic strain (*Lactobacillus rhamnosus* GG). Data showed a consistent activity pattern with previous assays on these enzymes. A crucial aspect addressed was the matrix effect, i.e., the impact of the growth media of the BSH-containing strains on biosensor output. This assay is expected to be a reproducible and accessible option, compatible with automated protocols.

## 1. Introduction

Bile acids (BAs) are fundamental molecules involved in the digestion and absorption of lipids and fat-soluble vitamins in humans and animals [1]. They are synthesized in the liver from cholesterol, secreted into the bile, and then they reach the intestine, where they act as natural detergents thanks to their amphipathic chemical structure [2]. BAs also function as signaling molecules interacting with receptors such as FXR and TGR5, regulating metabolic, immune, and microbiota-related processes [3,4]. The physiological pool of BAs in the human organism comprises primary bile acids, cholic acid (CA) and chenodeoxycholic acid (CDCA), which are conjugated with glycine or taurine to increase solubility and reduce toxicity. Once in the intestine, conjugated primary BAs undergo bacteria-mediated transformations that produce unconjugated forms, and subsequently, secondary BAs such as deoxycholic acid (DCA) and lithocholic acid (LCA). Each category of BAs has distinct physicochemical and biological properties. This metabolic flexibility is central to the enterohepatic circulation, whereby over 90% of BAs are reabsorbed and returned to the liver [5,6].

The gut microbiota plays a key role in these transformations, primarily through the action of bile salt hydrolase enzymes (BSH, EC 3.5.1.24) [7]. They catalyze the hydrolysis of the amide bond between bile acids and their conjugated amino acids [8]. BSH activity represents the first and essential step in microbial modification of bile acids, enabling downstream reactions such as 7α-dehydroxylation and epimerization that generate bioactive secondary BAs [9,10]. The presence and activity of BSHs are not only critical for microbial resistance to bile toxicity but also influence host physiology, affecting lipid metabolism, inflammatory responses, and intestinal barrier function. In this context, BSHs are considered key molecular markers in the evaluation of probiotic strains [11]. High-quality probiotics often exhibit robust BSH activity, which is associated with benefits such as cholesterol lowering, modulation of gut microbiota composition, and improved gut colonization [12]. Dysregulation of microbial-mediated BA metabolism is associated with the occurrence of pathological states in humans and animals. For instance, a change in the BA pool in the intestine from unconjugated and secondary BAs to conjugated types promotes the germination of previously *Clostridioides difficile* spores, and triggers *C. difficile* infection [13,14].

Heterologously expressed BSH enzymes were recently adopted as a pathogen inhibition strategy against *Clostridium perfringens* [15], relevant for animal infection, or *C. difficile* [16], for which the potential of an engineered BSH-producing probiotic was also assessed in a preclinical model. However, the uncontrolled production of certain secondary bile acids, such as LCA, has been linked to colorectal cancer, liver disease, and the recurrence of *C. difficile* infection, underlining the importance of precisely characterizing and regulating BSH functionality [17,18].

To identify and characterize BSH activity in microbes, assays can rely on the measurement of specific types of BAs, acting as reaction substrates or products, or by quantifying the amino acids liberated from conjugated BAs. Traditional quantification methods of BSH activity include chromatographic techniques (TLC, HPLC) and enzymatic colorimetric tests [19,20,21,22,23,24]. While these approaches offer precision and sensitivity, they are often expensive, time-consuming, and require specialized instrumentation, limiting their applicability in routine screening or large-scale testing [25]. Plate-based screening methods are also used as simple and low-cost assays, but provide only qualitative or semi-quantitative results, and have a lower sensitivity than chromatographic techniques [12,19]. In recent years, BSH characterization approaches based on synthetic probes have emerged as promising alternatives to traditional biochemical methods [25,26,27]. However, despite involving low-cost and user-friendly procedures, they require the synthesis of specific reagents.

The approach proposed in this work relies on an assay based on whole-cell biosensors, which are engineered living cells capable of detecting specific molecules and producing a measurable response [28,29], i.e., fluorescence as a function of primary conjugated BAs. Whole-cell biosensors combine genetic sensing modules (typically a regulated promoter and a reporter gene) with microbial chassis to transduce a chemical signal [30,31,32]. Compared to traditional assays, biosensor-based tests offer several advantages, such as their cost-effectiveness, scalability, and compatibility with high-throughput formats. Biosensor systems have been tailored to detect BAs using wild-type or engineered transcriptional regulators [33,34,35,36]. Despite showing different preference patterns for the diverse BA types, all of them were reported to sense multiple BAs. Among them, the TcpPH system from *Vibrio cholerae* was used in this study. It was previously engineered via the EMeRALD system [37] to obtain a transmembrane chimeric receptor for BAs, activated via ligand-induced dimerization, with a preference for the primary conjugated form [34]. This biosensor was successfully used for detecting BAs in real samples, e.g., clinical serum and fecal samples [34,38]. Biosensor output correlated with the total BA content of the samples, although a specific quantification of individual BA types could not be carried out, and matrix effect in fecal samples was observed. Such an effect, also addressed in this work, is a critical aspect in biosensor operating protocols and needs to be compensated for or characterized when dealing with real samples.

This work aimed to adopt the TcpPH-based biosensor for a novel application, regarding the identification and characterization of microbes with BSH activity, by quantifying the drop in conjugated BAs over time in cultures of BSH-containing strains. Its ultimate goal is to establish a reliable, sensitive, and accessible method to qualitatively and quantitatively assess BSH activity in bacterial strains, including potential probiotics.

## 2. Materials and Methods

### 2.1. Strains and Plasmids

All the strains used in this work are listed in Table 1. The TcpPH-based biosensor plasmid expressing GFP (Tcp-EMeRALD), harboring a chloramphenicol resistance and a p15A replication origin [34], was a gift from Dr. Jerome Bonnet (University of Montpellier, France). The plasmid was purified from recombinant DH10b cultures and transformed into the TOP10 strain, together with a second plasmid (BBa_J107125-pUC19), which was replicated thanks to a compatible origin (mutated pMB1), and provided the ampicillin resistance. The resulting recombinant strain (TcpSens) was used in this work as the BA biosensor strain, with chloramphenicol and ampicillin resistance. The EC-BSH-B and EC-BSH-C *E. coli* strains, containing expression plasmids for the BSH-B and BSH-C genes of *Lactobacillus johnsonii PF01* [20,39], were gifts from Prof. Dae-Kyung Kang (Dankook University, Republic of Korea).

*E. coli* cultures were prepared by inoculating 5 mL of LB (1% sodium chloride, 1% tryptone, 0.5% yeast extract) with single colonies and incubating them at 37 °C, 220 rpm overnight. Plasmid extraction was carried out with the NucleoSpin Plasmid kit (Macherey-Nagel, Düren, Germany), according to the manufacturer’s instructions. Transformation was carried out in competent TOP10 cells by heat shock at 42 °C, using purified plasmids. Long-term stocks of all the bacterial strains were prepared by mixing 750 µL of a saturated culture with 250 µL of 80% glycerol and were stored at −80 °C. Single colonies were obtained by streaking a long-term stock on an LB plate (1.5% agar) and incubating it at 37 °C overnight. *Lactobacillus rhamnosus GG* (LGG, ATCC 53103) was retrieved from a capsule of Kaleidon (Menarini Group, Florence, Italy), resuspended in sterile PBS, streaked on an MRS plate with 1.5% agar (#610024, Liofilchem, Roseto degli Abruzzi, Italy), and incubated at 30 °C for two days. A single colony was used to inoculate 5 mL of MRS. The culture was incubated at 30 °C, 220 rpm overnight, and finally, a long-term stock was prepared as described above.

### 2.2. Reagents

Chloramphenicol and ampicillin were prepared as sterile 34 mg/mL and 100 mg/mL stocks and used in selective media at the working concentrations of 12.5 µg/mL and 100 µg/mL, respectively. Isopropyl-β-D-1-thiogalactopyranoside (IPTG, #I1284, Sigma Aldrich, St. Louis, MO, USA) and antibiotics were routinely stored at −20 °C. Cholic acid (CA, #C1129), taurocholic acid (TCA, #T4009), chenodeoxycholic acid (CDCA, #C9377), glycochenodeoxycholic acid (GCDCA, #G0759), and taurochenodeoxycholic acid (TCDCA, #T6260) were purchased from Sigma Aldrich. Bile acid stocks were prepared as 100 mM solutions and routinely stored at −80 °C. The solvents used to prepare these solutions were deionized water (for TCA, GCDCA, and TCDCA), ethanol (for CDCA), and methanol (for CA).

### 2.3. Biosensor Characterization

Single colonies of the TcpSens strain were used to inoculate 0.5 mL of selective M9 medium (11.28 g/L of M9 salts—#M6030 Sigma Aldrich, 2 mM of MgSO_4_, 0.1 mM of CaCl_2_, 1 mM of thiamine hydrochloride, and 0.4% *v*/*v* of glycerol as the main carbon source), supplemented with 0.2% *w*/*v* of casamino acids, in a 2-mL tube. Cultures were incubated at 37 °C, 220 rpm overnight. The following day, 1-mL cultures including 890 µL of fresh medium, 10 µL of grown culture, and 100 µL of BA-containing samples at the desired concentrations were prepared and incubated under the same conditions as above for 6.5 h. Cultures were then centrifuged (10 min, 10,000 rpm, at room temperature, Biofuge Pico, Heraeus, Hanau, Germany), the supernatant discarded, and the pellet was resuspended with 1 mL of PBS. A 200-µL aliquot was transferred into a 96-well plate, and absorbance (optical density at 600 nm—OD_600_) and fluorescence (485 nm excitation, 540 nm emission, gain at 50) were measured in an Infinite F200Pro reader (Tecan, Männedorf, Switzerland).

### 2.4. BSH Activity Quantification

Assays were carried out as described in Section 2.3, with the exception that the BA-containing samples were obtained from bacterial cultures of strains bearing BSH enzymes, prepared as follows. BSH-containing strains were grown in 15-mL tubes, in 3 mL of M9 supplemented medium with ampicillin and 5 mM IPTG to trigger BSH expression (EC-BSH-B and EC-BSH-C strains) or 6 mL of MRS medium (LGG strain), which were inoculated with a single colony from an agar plate. When indicated, 0.2% glucose was added to MRS. At the beginning of the growth (t = 0), the specified BA was added at a 100 µM concentration. These cultures were grown at 37 °C (EC-BSH-B and EC-BSH-C) or 30 °C (LGG), 220 rpm for 24 h. Samples (200 µL) were withdrawn at the indicated time points, centrifuged (1 min, 13,000 rpm), and the supernatant was stored at −20 °C until further analysis. In every experiment with BA-containing samples at unknown concentrations, a calibration curve was constructed by inducing the TcpSens strain with BA samples at known concentrations, prepared in M9 supplemented medium or MRS (fresh or from a 24-h culture without BA, as indicated in the results).

**Figure 1:**
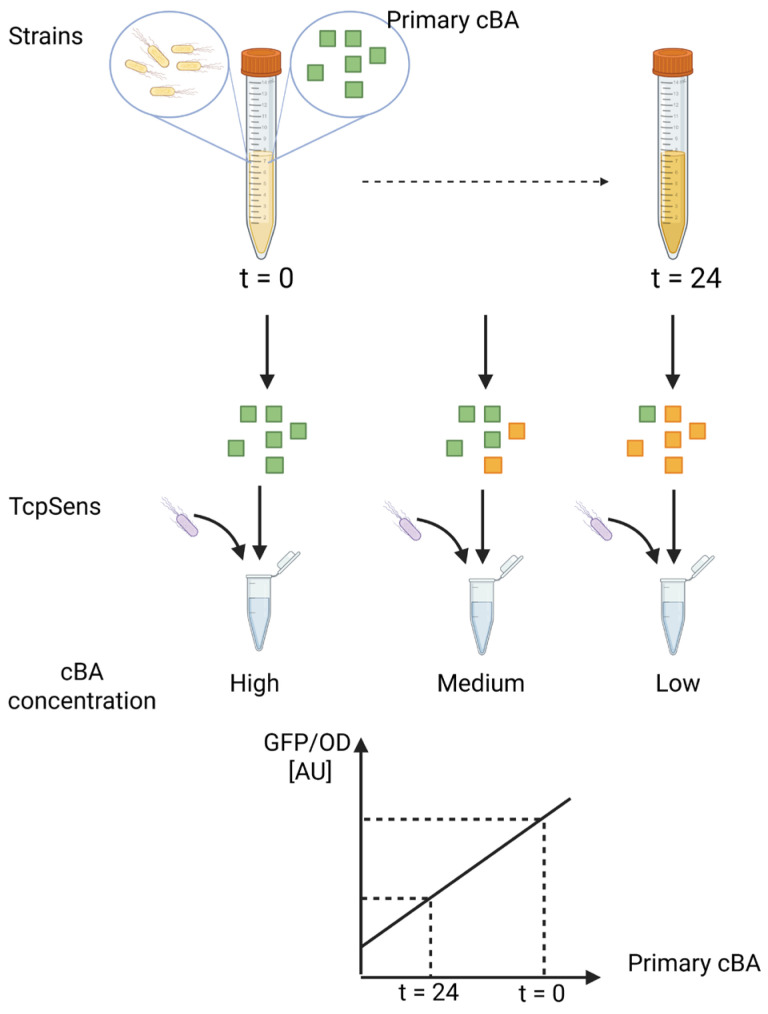
Schematic representation of the bile salt hydrolase (BSH) activity assay. The procedure involves culturing a BSH-expressing strain in the presence of a specific conjugated BA, withdrawing supernatant samples over time, and incubating them with a BA-responsive whole-cell biosensor (TcpSens). The biosensor per-cell fluorescence (GFP/OD) is used to determine the residual concentration of cBA via a calibration curve. Green squares, conjugated BA; yellow squares, unconjugated BA; yellow bacteria, target strain to be tested for BSH activity; grey bacteria, whole-cell biosensor; cBA, conjugated bile acid.

An illustration of the defined assay is provided as Figure 1.

### 2.5. Data Processing

Raw absorbance and fluorescence were background-subtracted using sterile media and a non-fluorescent culture, respectively, which were included in each test. The output of the TcpSens strain was quantified in terms of per-cell GFP fluorescence (GFP/OD) by dividing the background-subtracted GFP by the background-subtracted OD_600_. Calibration curves that relate GFP/OD (in arbitrary units—AU) and BA concentration were fitted with Hill equations (Equation (1)):

(1) $$\frac{GFP}{OD}=\delta +\frac{\alpha }{1+{\left(\frac{\kappa }{\left[BA\right]}\right)}^{\eta }}$$

where δ, α, κ, η, and [BA] are the basic activity in the absence of BA, the maximum activation range of the sensor, the half-induction concentration, and the concentration of BA, respectively. From the parametrized function of Equation (1), i.e., by estimating the δ, α, κ, and η parameters from calibration curves, it was possible to compute the unknown BA concentration of a new sample given its GFP/OD value, by simply inverting Equation (1) (see Equation (2)):

(2) $$\left[BA\right]=\kappa \cdot {\left(\frac{\alpha *\delta -\frac{GFP}{OD}}{\frac{GFP}{OD}-\alpha }\right)}^{\eta }\cdot d$$

where d is the dilution factor applied to the sample, which was normally 10, as the assay involves the addition of 100 µL of sample to 900 µL of culture (see Section 2.3). Unless otherwise indicated, in BSH characterization tests, the BA concentrations were expressed as a percentage of their initial values.

Data were processed via MATLAB 2023b (MathWorks, Natick, MA, USA) and Microsoft Excel. Graphpad Prism 10 was used for data visualization.

### 2.6. Mathematical Modeling

Mathematical models were defined (i) to fit the time course data of BA transformations to estimate the activity of BSH, and (ii) to investigate the effect of unspecific sensor activation in the presence of deconjugated BAs by a simulation framework.

#### 2.6.1. Dynamic Model of BA Deconjugation

An ordinary differential equation (ODE) model was defined to capture the deconjugation dynamics of BAs due to BSH during the growth of a BSH-expressing microbe (Equation (3)):

(3) $$\frac{d[BA(t)]}{dt}=-k_{BSH}\cdot [BA\left(t\right)]\cdot X(t)$$

where X is the bacterial concentration in the culture and k_BSH_ (min^−1^ cell^−1^) is the per-cell activity of BSH. By using OD_600_ as a measurement of cell concentration and the conjugated [BA] from biosensing assays, k_BSH_ was estimated in proportional units from four OD_600_ data points and four [BA] data points. In particular, it was estimated by solving and fitting Equation (3) using the MATLAB *lsqnonlin* and *ode23s* routines, respectively. OD_600_ time course was provided to the ODE solver as a shape-preserving piecewise cubic interpolation by the *interp1* routine using the *pchip* option.

#### 2.6.2. A Simulation Model to Study the Impact of Biosensor Unspecificity

A two-equation system was defined to simulate a BA-transformation experiment in which BSH converts a conjugated BA (cBA) into its deconjugated form (uBA). During BSH assays, a known amount of cBA (e.g., TCDCA) is added to the culture, and it is transformed over time into uBA (e.g., CDCA). This system was derived from Equation (4). cBA and uBA are assumed to strongly and weakly activate the biosensor, respectively. This simulation framework aimed to assess whether the fluorescence coming from CDCA (uBA) could strongly affect the quantification of TCDCA (cBA) deconjugation by the biosensor.


(4) $$\left\{\begin{array}{c} F_{c}=\delta +\frac{\alpha_{c}}{1+\frac{\kappa_{c}}{\left[cBA\right]}}\\ F_{u}=\delta +\frac{\alpha_{u}}{1+\frac{\kappa_{u}}{\left[uBA\right]}} \end{array}\right.$$

The F symbols are the GFP/OD output of the biosensor in the presence of a specific BA (cBA or uBA), and the parameters have the same meaning as in Equation (1). Under the assumptions that: the calibration curve is fitted sufficiently well for η = 1, no measurement error affects fluorescence, the dose–response curves are not in the saturation regime, and two BAs additively affect the output of the biosensor, Equation (4) becomes linear and can be rewritten as Equation (5):

(5) $$\left\{\begin{array}{c} F_{c}=\delta +m_{c}\cdot [cBA]\\ F_{u}=\delta +m_{u}\cdot [uBA]\\ F_{tot}=F_{c}+F_{u}\\ {\left[cBA\right]}_{t=0}={\left[cBA\right]}_{t}+{\left[uBA\right]}_{t} \end{array}\right.$$

where F_tot_ is the output of the sensor for each combination of cBA and uBA.

The model was parametrized based on typical experiments with TCDCA as cBA and CDCA as uBA, in which the initial concentration of TCDCA was 10 µM and the slopes m_c_ and m_u_ were estimated by linear regression of dose–response curves with TCDCA and CDCA, respectively. From the obtained model, it is possible to simulate the contribution of [cBA] and [uBA] on GFP/OD. As GFP/OD is used to measure the concentration of cBA, based on Equation (2), the typical error in cBA quantification due to unspecific sensor activation by uBA can be finally simulated.

### 2.7. Statistical Analyses

Statistical tests were used (i) to identify the limits of detection (LODs) of the biosensor to different BAs by one-tailed *t*-tests for independent samples, (ii) to compare the measured BA concentrations between t = 0 and t = 24 h by one-tailed *t*-tests for paired samples, and (iii) to compare the biosensor output in calibration curves constructed with different matrices, at a given BA concentration, by two-tailed *t*-tests for independent samples. Tests were performed by Graphpad Prism 10, in which a *p*-value (*p*) cutoff of 0.05 was used.

In the biosensor characterization part, data replicates refer to biological replicates carried out in independent tests that started from different colonies of TcpSens. Their value was expressed in terms of fold-change with respect to the output in the absence of BA, measured in the same experiment. Their variability was expressed as the standard error of the mean.

In the BSH assays, replicates refer to biological replicates from independent experiments in which different bacterial cultures producing BSH were analyzed, and their variability was expressed as standard deviation. When indicated, the variability of OD_600_ measurements in these assays was expressed as a 95% confidence interval of the mean value.

Relative error was used to quantify the deviation in BA values measured in sensing assays from the true values of spiked BA samples. The number of replicates is indicated in the figures or in the text.

Finally, to quantify the minimum BA decrease (Δ_min_) that can be detected as statistically significant in a BSH assay, Equation (6) was used, based on a one-tailed *t*-test for paired samples:

(6) $$\Delta_{min}=\frac{t_{0.95,N-1}\cdot s_{d}^{*}}{\sqrt{N}}$$

where N is the number of biological replicates, $s_{d}^{*}$ is the typical standard deviation of the differences between initial and final measured concentration of conjugated BA, and *t*_0.95,N−1_ is the Student’s *t* distribution value that corresponds to an area under the curve of 0.95, with N − 1 degrees of freedom. The value of $s_{d}^{*}$ was computed as the mean of the standard deviations of BA decrease in four conditions in which BSH activity was significant: EC-BSH-B with TCA, EC-BSH-B with TCDCA, LGG with GCA in MRS medium, and in MRS medium with glucose.

## 3. Results

### 3.1. Biosensor Selection and Characterization

First, a proper whole-cell biosensor was selected among the available ones to eventually define a sensing assay. The main specifications were to have an inducible genetic system in which the expression of a reporter gene could be triggered by primary conjugated bile acids (BAs), using a non-pathogenic and fast-growing host as a chassis. The synthetic TcpPH-EMeRALD system, involving an engineered tcpP-cadC gene and the tcpH gene from the TcpPH system of *V. cholerae*, was selected due to its preference for the primary conjugated forms [34]. This artificial system enables the activation of the P_cad_ promoter in *Escherichia coli* in the presence of specific BA types. Other BA-responsive systems were considered, for which synthetic circuits were constructed, and data are available in the literature. They were VtrAC-EMeRALD (based on the *Vibrio parahaemolyticus* VtrAC system [34]), CmeR (from *Campylobacter jejuni* [40]), and VFA0359 (from *Alivibrio fischeri* [35]). These systems were excluded due to a lack of specificity for the desired BA types (VtrAC [34] and CmeR [41,42]), or because they were implemented in anaerobic hosts, making user-friendly assays impractical (VFA0359 [35]). The BreR system from *V. cholerae* was also recently reported, but its activation pattern towards primary BAs was found to be close to that of TcpPH [33] and was therefore not considered further.

The sensing assay involves adding a supernatant sample from a BA-transforming microbe into a biosensor strain culture, triggering GFP expression. Since the present study also includes recombinant BSH strains, grown in selective conditions with an antibiotic, the biosensor strain was equipped with a plasmid conferring ampicillin resistance to allow for sensing assays with antibiotic-containing supernatants. Similar strategies were previously adopted [43] and should be considered in the future definition of other biosensor-based procedures to limit matrix-borne inhibition effects.

**Figure 2:**
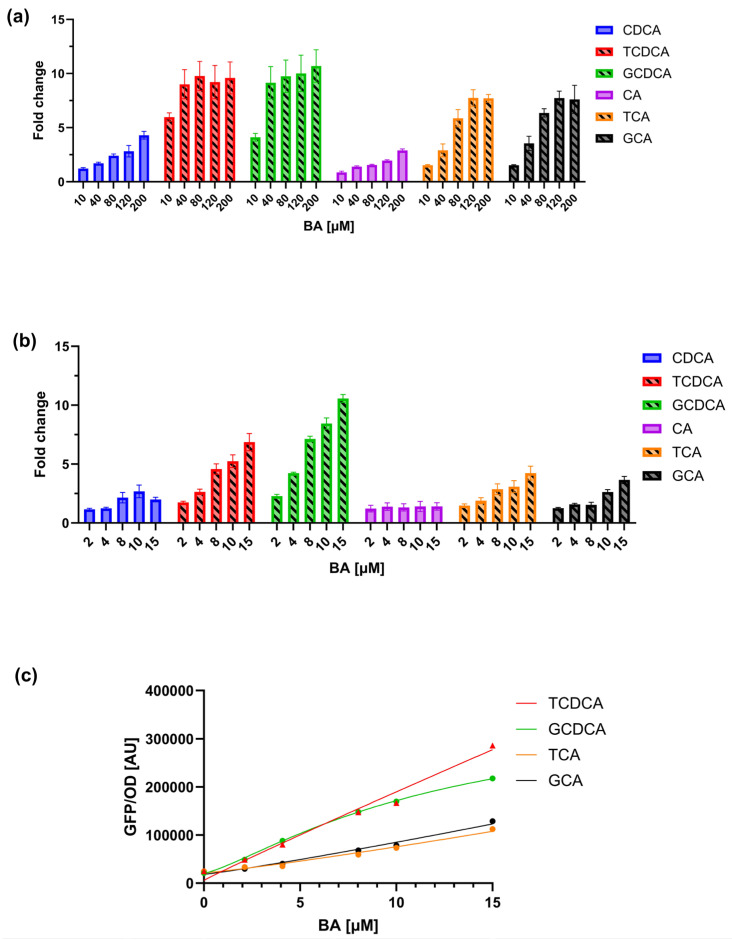
Characterization of the TcpSens biosensor response to different primary BAs. (**a**) Dose–response curves showing the GFP/OD output of TcpSens in the presence of increasing concentrations (0–200 µM) of various BAs. Bars represent the average values of at least three independent measurements, and error bars represent the standard error of the mean. (**b**) Response in the 0–10 µM range for conjugated and unconjugated BAs, showing a reduced response to the unconjugated forms and a more linear response to conjugated BAs. Bars represent the average values of at least three independent measurements, and error bars represent the standard error of the mean. Bars with diagonal hatching and solid bars indicate conjugated and unconjugated BAs, respectively. (**c**) Representative calibration curves fitted by Hill equations. Parameter estimates (α, AU; κ, µM; η, -; and δ, AU) were: α = 6.47 × 10^7^, κ = 4.36 × 10^3^, η = 0.96, δ = 5.78 × 10^3^ (for TCDCA); α = 3.45 × 10^5^, κ = 12.15, η = 1.30, δ = 2.07 · 10^4^ (for GCDCA); α = 2.27 × 10^7^, κ = 1.90 × 10^3^, η = 1.15, δ = 2.15 × 10^4^ (for TCA); α = 3.10 × 10^7^, κ = 2.46 × 10^3^, η = 1.12, δ = 1.84 × 10^4^ (for GCA).

To assess the sensitivity and specificity of the biosensor strain (TcpSens) towards conjugated BAs, dose–response curves were measured in terms of GFP/OD for six different BAs, including primary unconjugated BAs (cholic acid—CA, and chenodeoxycholic acid—CDCA) and primary conjugated BAs (taurocholic acid—TCA, glycocholic acid—GCA, taurochenodeoxycholic acid—TCDCA, and glycochenodeoxycholic acid—GCDCA), spanning a wide range of molecule types with relevance for human and animal metabolism. Data were first collected in the 0–200 µM concentration range for all six BAs (Figure 2a), corresponding to a non-toxic range in *E. coli* for all the molecules (data not shown). Data showed that all the BA types could trigger a response, with activation fold-changes (maximum output divided by the output in the absence of BA) ranging from 2.5-fold for CA to 10-fold for TCDCA and GCDCA. The preference pattern is consistent with the characterization that was reported in [34] but highlights that unspecific activations can occur by non-conjugated BA forms in the tested range.

### 3.2. Definition of the Biosensor-Based Assay

The designed sensing assay is described in Section 2.4. It was defined to measure the deconjugation capability of a cultured strain, from which samples are withdrawn over time and added to a culture of the biosensor. It will provide a fluorescent output that is related to the unknown concentration of conjugated BA, according to a calibration curve. The conjugated BA is expected to decrease over time, and its unconjugated form is expected to make a negligible contribution to the output fluorescence (Figure 1).

In this assay, 100 µM of conjugated BA was added to a culture of the target strain to be characterized in terms of BSH activity. The concentration window of conjugated BA during strain growth will be 0–100 µM, resulting in a 0–10 µM range in the biosensor culture due to the 10-fold dilution of the sample (Equation (2)). This dilution could be theoretically decreased to improve sensitivity, as previously conducted in other biosensor assays [43,44], but in this work, it was kept constant. In the 0–10 µM BA range, the benefits are twofold, as demonstrated by an additional characterization carried out in this input range (Figure 2b). First, the biosensor has a relatively linear output in this range, which facilitates the calculation of BAs from calibration curves that will not show saturations. As a result, the maximum fold-activation for the conjugated BAs in this range is systematically lower than in Section 3.1, from 3.6-fold (GCA) to 10.6-fold (GCDCA). Second, the biosensor output in response to unconjugated BAs, which trigger an unwanted fluorescent signal, was minimal: for all the tested CA concentrations in this range, it was not statistically different from the non-induced condition, and it decreased to only 2-fold for CDCA. The conjugated BA-GFP/OD characteristics, which will serve as calibration curves, could be fitted well by Hill equations (Figure 2c), as previously reported [28,34]. Using a narrow concentration range of BAs, the estimated parameter values are useful to capture the experimental data and enable the measurement of unknown BAs in biosensor assays by Equation (2). This approach does not guarantee a robust Hill model identification, as dose–response curves are in their linear range and saturation is not observed, making the estimation of model parameters difficult. Nonetheless, the obtained calibration curves are considered reliable if they are not used to extrapolate values outside the curve range (i.e., 2–15 µM). Other mathematical functions, like linear models, polynomials, or splines, could be used to fit calibration data. However, the construction of calibration curves with a function that is already known to accurately fit these data was preferred, even though no relevant changes in quantifications are expected if different mathematical functions that well-fit the data are used.

**Figure 3:**
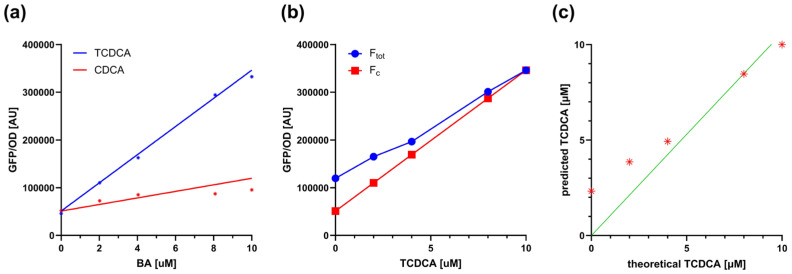
Modeling the contribution of unconjugated BAs to biosensor output during deconjugation. (**a**) Calibration curves of TcpSens for TCDCA (conjugated) and CDCA (unconjugated), fitted with the linear model of Equation (5). Fitted parameters were m_c_ = 2.95 × 10^4^ AU µM^−1^, m_u_ = 6.86 × 10^3^ AU µM^−1^, and q = 2.56 × 10^4^ AU. Data points come from a single representative experiment, and the solid lines represent the fitted linear model. (**b**) Simulation of fluorescence output resulting from mixtures of TCDCA and CDCA at fixed total BA concentration (10 µM). The F_tot_ and F_c_ values (see Equation (5)) are reported as a function of TCDCA. Circles and squares represent simulated fluorescence outputs, as indicated in the legend, while solid lines represent linear interpolation. (**c**) Theoretical concentration of TCDCA and its predicted quantification in the presence of non-specific biosensor activation by CDCA. Asterisks indicate the simulated values, while the solid line represents the bisector line.

Considering that the conjugated forms of CDCA (i.e., TCDCA and GCDCA) induce TcpSens up to 6.9- and 10.6-fold, respectively, the contribution of CDCA to fluorescence is expected to be small. To quantitatively understand if this unwanted contribution is relevant in the sensing assay, a mathematical model was defined, describing the fluorescence output of the biosensor in the simultaneous presence of a conjugated BA and its non-conjugated form (see Section 2.6). The worst case of non-specific response described above was considered, i.e., the TCDCA/CDCA pair, in which TCDCA is converted into CDCA by BSH. As a result, the total TCDCA + CDCA concentration is kept constant and equal to 10 µM. The model of Section 2.6 was parametrized by estimating calibration curves from real data (Figure 3a). For the sake of simplicity of the downstream calculations, the calibration curves were fitted by linear models instead of Hill equations, as in the standard assays, capturing the data reasonably well. The model was first used to compute the fluorescence output for different combinations of TCDCA and CDCA (Figure 3b); then, the calculated fluorescence and the calibration curve of TCDCA were used to compute the concentration of TCDCA, mimicking a real assay in which a small part of fluorescence due to CDCA is present but neglected in the calibrations (Figure 3c). A comparison between computed and actual TCDCA showed that an overestimation is expected for small concentrations of TCDCA. In particular, the relative error remains low (<25%) for concentrations as low as 4 µM, even though the concentration of CDCA becomes larger than that of TCDCA. The deviation increases for TCDCA concentrations around 2 µM by about 2-fold, reaching an estimated TCDCA value of 2.3 µM when all the conjugated form is transformed (Figure 3c). Nonetheless, if the scope of the assay is to screen probiotics for their BSH activity, the result is expected to be sufficiently accurate to discriminate BSH-positive and BSH-negative strains and also to provide a ranking of their activities. In fact, this issue would be present only for assays on TCDCA or GCDCA with strains having a high BSH activity, where a nearly complete transformation of conjugated forms of CDCA (higher than 60%) would result in an underestimation of the conversion capability, but still maintaining the BA ranking of different conditions.

Using the data of Figure 2b, biosensor performance was then analyzed in terms of limit of detection (LOD), i.e., the smallest concentration of the calibration curves that results in a statistically higher fluorescence compared to the non-induced condition. This BA concentration in the calibration curve was 2 µM (*p* < 0.05, *t*-test), resulting in a LOD of 20 µM due to the 10-fold dilution applied to the samples. This corresponded to an 80% conversion from conjugated to unconjugated BA. The reproducibility of the assay was finally analyzed by quantifying different M9 supplemented media samples spiked with BAs (TCA and TCDCA used as a testbed). The average relative error between measured and true values was 26% (N = 15), which is considered reproducible, based on other synthetic biology-based biosensors that showed variability up to ~20% [43,45].

In the following sections, the assay was then evaluated in the characterization of three strains bearing BSH enzymes.

### 3.3. BSH Activity Characterization in Recombinant Strains

The sensing assay was first evaluated on two recombinant strains of *E. coli*, bearing expression plasmids with distinct BSH enzymes (BSH-B and BSH-C) from *L. johnsonii* PF01. Calibration curves, constructed by exposing TcpSens to known concentrations of BAs, showed no difference between the use of fresh M9 supplemented medium or spent medium from a 24-h saturated culture in the presence of the same BA concentrations (data not shown). This demonstrates that no matrix effect occurs for this medium and enables us to use the same calibration curve to quantify samples taken from t = 0 and t = 24 h cultures.

**Figure 4:**
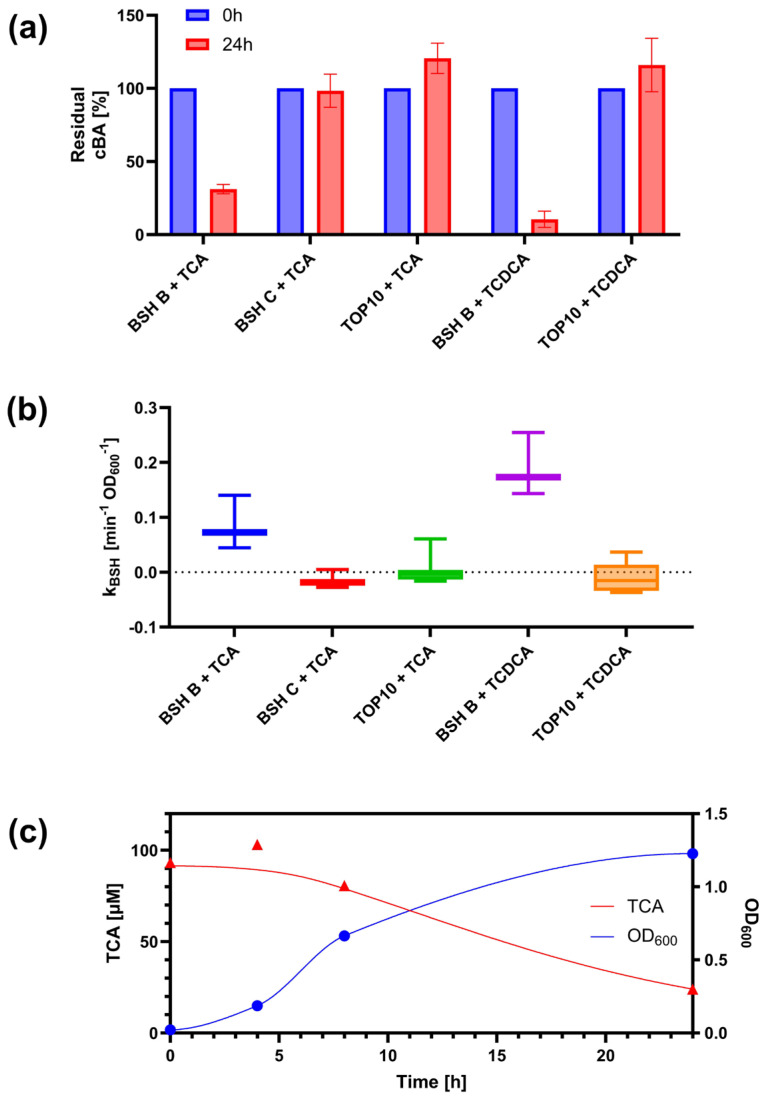
Detection of BSH activity in recombinant *E. coli* strains. (**a**) Residual concentrations of conjugated BAs (cBA) after 24 h in cultures of EC-BSH-B, EC-BSH-C, and no-BSH control (NC) strains. Data points represent the average values of at least three independent experiments, and error bars represent the standard error of the mean. (**b**) Time course of cell growth and BA deconjugation in EC-BSH-B cultures with TCA. Data points represent the experimental measurements of a representative test. Solid lines represent the fitted concentration profile of TCA using Equation (3) and the interpolation line for OD_600_, respectively. (**c**) Estimated BSH activity per cell density (k_a_) by fitting deconjugation data with the model of Equation (3). Circles and triangles represent OD_600_ and TCA data, respectively, and solid lines interpolation (OD_600_) or fitting (TCA), with the colors indicated in the legend. Boxplots indicate the distribution of k_a_ values in at least three independent tests. The labels in panels (**a**,**c**) indicate the strain and the initially added BA (TCA or TCDCA).

The assay defined in this work was expected to detect the deconjugation activity of the strains under investigation, according to the preferences of the two enzymes for specific substrates, previously characterized in vitro [20,39]. For BSH-B, the biosensor successfully detected a decrease in tauroconjugated BAs (TCA and TCDCA), initially added to the BSH-expressing recombinant cultures (Figure 4a). This decrease was statistically significant (*p* < 0.05, *t*-test) and accounted for a 69% and 90% reduction in TCA and TCDCA, respectively. Conversely, EC-BSH-C did not exhibit deconjugation activity against TCA and TCDCA, as the initially added BA did not decrease over the 24-h experiment (*p* > 0.05, *t*-test).

The detected deconjugation pattern matches the expected preferences of the two enzymes, with BSH-B having a high activity against tauroconjugated BAs, and BSH-C having a poor activity with the same substrates. The preference was expected to be the opposite for glycoconjugated BAs. However, the biosensor assay showed no detectable deconjugation activity for both BSH-B and BSH-C (data not shown). The undetectable activity in EC-BSH-C cultures may be due to the non-optimal conditions for BSH-C expression and activity in terms of growth medium and oxygen. BSH-C activity was previously demonstrated in purified enzyme, with an optimal temperature of 70 °C, and in anaerobic LB agar plates [39], while in the present work, the use of a more nutrient-poor medium and the oxygen availability in liquid cultures may represent a less favorable condition. For this reason, the data obtained from EC-BSH-C were not considered indicative of BSH-C activity patterns.

A bacterial culture without BSH activity (TOP10 strain of *E. coli*) was always included in the experiments as a negative control. Importantly, this culture showed no deconjugation, as the concentration of the initially included BA at the beginning of the growth was comparable to the measured BA level after 24 h (*p* > 0.05, *t*-test), assessing the specificity of the assay (Figure 4a).

Finally, the defined assay was used to characterize the BSH-B deconjugation activity in a time course experiment, monitoring cell growth and conjugated BA. As expected, data showed a decrease in TCA and TCDCA in the EC-BSH-B culture, which increased in density (Figure 4b). The temporal profile of BA could be fitted with the dynamic model of deconjugation activity presented above (Equation (3)), to estimate the unknown parameter describing the per-cell deconjugation rate, considering cell growth in the analysis of BSH-containing strains (Figure 4b,c).

Altogether, these data demonstrated the feasibility of monitoring the deconjugation capability of BSH-containing strains via a simple fluorimetric assay. Next, we aimed to demonstrate that BSH activity could be detected in a real scenario, in a probiotic strain.

### 3.4. BSH Activity Detection in a Probiotic Strain

The second evaluation of the designed sensing assay was on *L. rhamnosus* GG (LGG), which is commercially used as a probiotic strain due to its beneficial features. Among these features, a BSH activity against glycoconjugated BAs was expected, whereas a lower activity was previously described against tauroconjugated BAs [12]. For this reason, tauroconjugated BAs were not tested in this work with LGG, and GCA was used as a representative glycoconjugated BA in the following tests.

**Figure 5:**
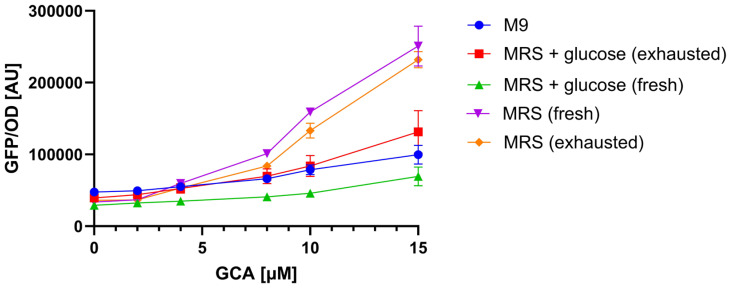
Matrix effect on TcpSens biosensor output. Calibration curves are constructed in different matrices (M9, MRS, MRS + glucose), using GCA as a testbed. The conditions with MRS and MRS + glucose were tested as fresh media and exhausted media from BA-free supernatants. Data show how the growth medium and the presence of glucose affect the biosensor response, highlighting the need for matrix-specific calibration curves. Data points represent the average values of at least three independent measurements, and error bars represent the standard error of the mean. The OD_600_ values for the illustrated conditions were (mean and standard deviation): 0.12 ± 0.03 (M9), 0.12 ± 0.04 (MRS + glucose, exhausted), 0.35 ± 0.05 (MRS + glucose, fresh), 0.12 ± 0.01 (MRS, fresh), 0.09 ± 0.01 (MRS, exhausted).

As the growth media of lactic acid bacteria are different from those of *E. coli* (used for TcpSens assay and growth of the BSH-containing strains of Section 3.3), calibration curves were first characterized by adding samples of MRS medium with known BA amounts (Figure 5). Data on GCA showed that the calibration curves with fresh and exhausted MRS samples were comparable (less than 1.2-fold changes, *p* > 0.05, *t*-test), but could reach up to 2.5-fold higher GFP/OD values than with M9 samples (*p* < 0.05, *t*-test). Unexpectedly, a relevant change in calibration curves was detected by adding samples of MRS with glucose (up to 3.6-fold lower than MRS without glucose, *p* < 0.05, *t*-test). This inhibition of the TcpSens activity by glucose was confirmed by analyzing BA samples prepared in exhausted MRS medium with glucose. As expected, the calibration curve was higher than with fresh MRS with glucose (up to 1.9-fold, *p* < 0.05, *t*-test), since a part of the sugar was most probably consumed, even though not completely, as the output values could not reach those of MRS without sugars.

The analysis above (Figure 5) demonstrated that calibration curves were sensitive to different media (e.g., M9 vs. MRS), but in one case also to the same medium at different time points (MRS with glucose). For this reason, calibration curves were constructed in each test with the medium used to cultivate the strains under investigation and, in the case of MRS with glucose, using both fresh and exhausted medium from a strain grown without BAs.

Sensing assay reproducibility was quantified as before (Section 3.2) using samples with known amounts of GCA, used as a testbed, in MRS or MRS with glucose to verify that the change of matrix does not deteriorate the performance of the assay. The average relative error between measured and true values was 35% (N = 9), which was slightly higher than in the previous analysis with M9 medium (Section 3.2).

**Figure 6:**
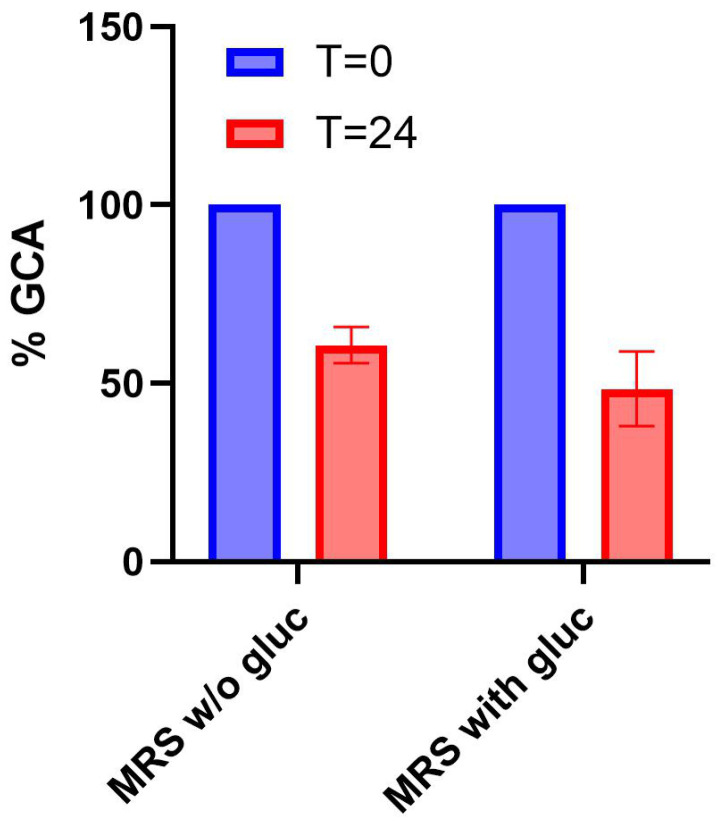
Detection of BSH activity in a probiotic strain. Residual concentration of GCA after 24 h in cultures of *Lactobacillus rhamnosus* GG (LGG) grown in MRS or MRS + glucose. Data points represent the average values of at least three independent experiments, and error bars represent the standard error of the mean.

Finally, samples from LGG cultures in MRS or MRS with glucose were analyzed (Figure 6). LGG reached a relatively low cell density in MRS without glucose (average OD_600_ of 0.2 ± 0.02 at t = 24 h), corresponding to a 12-fold lower growth than in MRS with glucose (average OD_600_ of 2.5 ± 0.05 at t = 24 h). All the measured cell density values were above the detection limit of the plate reader and were highly reproducible. Nonetheless, a statistically significant decrease in GCA was detected in both conditions (29% and 52%, respectively, *p* < 0.05, *t*-test). Considering the different cell density reached, the data suggest that the probiotic strain exhibits BSH activity in both the absence and presence of sugar, but its per-cell activity in media without glucose was higher.

Overall, data showed that the defined assay could also be applied to detect BSH activity in a naturally occurring strain that is relevant as a supplement for human health.

Based on the data gathered for the two strains that showed a significant BSH activity (EC-BSH-B and LGG), the typical standard deviation of the measured percent decreases in conjugated BAs among biological replicates (12.2%) was computed, as described in Section 2.7. Then, to give a quantification of the smallest decrease that could generate a statistically significant outcome (i.e., a significant BA decrease from the 100 µM baseline) and result in the identification of a BSH-positive strain, Equation (6) was used. This decrease is a function of the number of biological replicates (N). For N = 3, the measured decreases larger than ~20% are expected to be statistically significant (Table 2). By increasing N to 4 or 6, the minimum decrease becomes much smaller (~14% and 10%, respectively). These data provide a theoretical framework to inform operators on the minimum deconjugation activity that could be detected and eventually quantify the sensitivity of this assay.

## 4. Discussion

The BSH detection assay defined in this work has the potential to overcome limitations of current techniques, offering a user-friendly, cost-effective, and reproducible method to screen bacterial isolates for BSH activity. It bridges synthetic biology with microbial ecology and opens new avenues for the rational design of engineered probiotics capable of modulating host bile acid metabolism, as the screening of efficient BSH variants could become easier to perform. Ultimately, this biosensing toolkit may become a valuable asset for microbiome-based therapeutics, functional probiotic screening, and metabolic engineering applications aimed at fine-tuning bile acid profiles in health and disease states.

The core of the proposed assay is a whole-cell biosensor, which is not new, as it was constructed and optimized in a recent work [34]. Instead, its application to BSH screening is a novel aspect that could address some of the disadvantages of the existing methods.

Our data showed consistent BSH activity patterns for the considered case studies. Specifically, the defined assay detected the activity of recombinant BSH-B from *L. johnsonii* PF01 against tauroconjugated BAs and not against glycoconjugated forms. However, BSH-C activity against glycoconjugated BAs, reported in the literature, was not detectable. We attributed this inconsistency to the growth conditions used: M9 supplemented media in test tubes were used in this study, whereas previous reports showed BSH-C activity for the same strain in agar plate assays using a rich medium (LB) and incubating the plate anaerobically [39]. Nutrients are known to have an impact on recombinant expression, and BSH enzymes are known to be oxygen sensitive [15,23,24]. For these reasons, even though BSH-B showed a successful activity in the same culture conditions, the different expression, activity, and/or oxygen-sensitivity between the two enzymes may result, in our hands, in an undetectable activity for BSH-C. The third case study was *L. rhamnosus* GG, a commercial probiotic, which showed BSH activity against glycoconjugated BAs, confirming the potential of the defined assay in a real case study.

One of the limits found in the present study was a matrix effect, as the biosensor output quantitatively changed (up to 3.6-fold) as a function of the sample type, i.e., the growth media of the strains under investigation. Matrix effect is commonly found in many biosensor-based assays, including sensing procedures with a TcpPH-EMeRALD strain in fecal samples, similar to the biosensor used in this work [38]. Matrix effect was not observed in every sample type tested in this work: in fact, no difference in biosensor output was detected from fresh to exhausted M9 and MRS media, used to grow Gram-negative and lactic acid bacteria, respectively. Conversely, biosensor output increased in MRS compared with M9. Also, MRS supplemented with glucose resulted in a lower biosensor output compared with sugar-free MRS, and exhausted MRS with glucose resulted in an intermediate output, confirming that the presence of glucose inhibited the sensor response to BAs. The P_cad_ promoter is reported to be induced when pH is low (<6.8) and at high lysine concentrations (>1 mM of free amino acid, reaching a plateau at 10 mM) [46]. This naturally occurring activation could interfere with the artificially designed BA-sensing network. The lower pH of MRS (5.7 ± 0.1 according to the manufacturer datasheet) than M9 (about 6.9, experimentally measured) in the presence of about 1 mM of lysine (reported to be about 7.5% in casamino acids, which are present in the biosensor culture medium) may explain a slight increase in the output in MRS samples compared with M9 samples, although the biosensor medium retains a good buffering capability. Lysine content in MRS (about 0.5 mM [47]) is not sufficient to explain the activation of P_cad_. The presence of glucose may also provide a fermentable source for the biosensor strain, possibly lowering the pH over the 6.5-h incubation. However, a drop in pH is not compatible with a decrease in biosensor output, suggesting that other unreported effects may influence P_cad_ in the presence of glucose. The influence of carbon sources has also been reported in other biosensors, including BA-sensitive ones (e.g., glycerol affecting the CmeR system [40]). Overall, these data suggest that the characterization of matrices is a critical step. In this work, matrix effect could be fully compensated by the construction of proper calibration curves, obtained by measuring the biosensor response in the same matrices used during the assays. This step is feasible because the designed assay relies on cultured bacteria, for which a sample of the matrix could be easily obtained with and without the addition of BAs. However, different assays in less controlled conditions may not allow us to construct such calibration curves and would limit the applicability of biosensors showing strong crosstalk. In addition, although matrix effect was herein compensated, other growth media might affect the output of the biosensor by changing the LOD or even by completely inhibiting GFP production, therefore preventing the use of this assay.

The lowest BA concentration that could be statistically distinguished from zero by the analyzed biosensor in the M9 media was 2 µM. Based on the 10-fold sample dilution used in this work, the LOD of the sensor was then quantified as 20 µM. This means that BSH-positive strains with a high enzymatic activity deconjugating more than 80% of the initially added BA (100 µM) will result in residual BA measured under the LOD. These strains will be clearly recognized as BSH-positive microbes, but the BA decrease will be quantified as 100%, making small differences among microbes with high BSH activities indistinguishable. Other BSH detection methods directly rely on BA-sensing elements: TLC was reported to quantify deconjugated BA forms with a LOD of about 0.4 mM for CA [19]. Previous reports using HPLC techniques relied on quantifying conjugated or unconjugated BAs during BSH reactions [22]. Although both forms could be detected with typical LODs lower than 1 µM [48], HPLC-based BSH assays were reported to quantify the decrease in conjugated BA concentration (TCDCA and GCDCA) instead of an increase in CA due to the faster and simpler method [22].

Because of the widely different principles underlying the various types of BSH assays, the smallest detectable enzyme activity depends on many factors. For example, the LOD in TLC was sufficient to appreciate the deconjugation of 5% of the initially available conjugated BA pool (8 mM) from in vitro reactions [19]. HPLC is reported to be highly precise, with intra-day variabilities below 4%, suggesting sensitive detection of small decreases in conjugated BAs, not explicitly quantified [22,48]. Other assays relying on amino acid or fluorogenic/luminescent substrate release, which do not directly quantify BAs, are reported to provide a detectable output in response to BSH concentrations in the mid-nanomolar (e.g., assays with aminocoumarin fluorophore probes) to micromolar (e.g., ninhydrin assay) range in vitro [27]. The main advantages and disadvantages of these methods were summarized in Table 3. As an exhaustive comparison of their sensitivity in different types of samples is hard to perform due to the different experimental conditions and the lack of data in comparable units, in the future, large-scale systematic evaluations should be performed to provide a fair and exhaustive comparison.

To provide a quantitative indication of the sensitivity of the designed assay, a statistical framework was used to calculate the minimum decrease in conjugated BA concentration that results in a statistically significant deviation from the initially added BAs (t = 0). With three biological replicates, a 20% decrease in conjugated BA could be detected by the biosensor analyzed in this work. Differences below 15% could be measured by increasing the number of replicates to four. This means that the assay is expected to identify BSH-positive microbes for which the enzymatic activity exhibits an average decrease in conjugated BA of, e.g., 20% with three replicates, over a given growth period. A strategy to improve sensitivity could be to tune the growth conditions of the investigated strain in terms of inoculum size, to increase the bacterial biomass that is capable of BA transformation, thus reaching a faster deconjugation rate.

All the techniques of Table 3 aim to identify BSH-positive microbes, but only some of them are compatible with the measurement of deconjugation dynamics in live cultures: ninhydrin assay requires enzyme purification to remove interfering reactants from growth media; in probe-based methods, the synthetic BA analogs used in the assays might have different affinities and membrane permeability from natural BAs, even though some of them were successfully tested with cultured bacteria [25,26,27]. Among the methods compatible with deconjugation monitoring in live cultures, to our knowledge, agar plate assays are not used to study deconjugation time courses, and their sensitivity was demonstrated to be lower than TLC [12]. Moreover, a previous study failed to detect BSH activity against GCA in *L. rhamnosus* GG using agar plate assays [12], while it was successfully detected in this work in the same media, suggesting a superior sensitivity in the herein proposed method. TLC and HPLC can accurately measure substrates and/or products of BSH activity in samples taken from live cultures; however, they suffer from low to moderate throughput in data generation [12,27]. Conversely, the assay proposed in this work is compatible with automated protocols in high-throughput format that can be operated via liquid handling platforms. For this reason, it is expected to enable large-scale studies on candidate probiotic strains to (1) support the identification of BSH-positive microbes, and (2) dynamically observe their activity in specific media and growth conditions.

## Figures and Tables

**Table 1:** Strains used in this work.

Name	Host	Plasmids	Scope	Reference
NC	TOP10	-	Host strain, negative control in BSH assays	Invitrogen
Tcp-EMeRALD	DH10b	P_5_-tcpH-P_9_-cadC-tcpP-P_cad_-GFP	Source of biosensor plasmid	[34]
TcpSens	TOP10	P_5_-tcpH-P_9_-cadC-tcpP-P_cad_-GFP and BBa_J107125-pUC19 ^1^	Biosensor strain	This study
EC-BSH-B	BLR(DE3)	pET-BSH-B	*E. coli* with BSH-B from *L. johnsonii* PF01	[20]
EC-BSH-C	BLR(DE3)	pET-BSH-C	*E. coli* with BSH-C from *L. johnsonii* PF01	[39]
LGG	*L. rhamnosus* GG	-	Probiotic strain with BSH	Menarini Group

^1^ BBa_J107125 is an IPTG-inducible sgRNA expression cassette that is cloned into the pUC19 high-copy vector. In this work, it has been used as a source of ampicillin resistance, not for its CRISPR-related function. The sequence of the insert can be retrieved from the Registry of Standard Biological Parts (https://parts.igem.org, accessed on 21 October 2025).

**Table 2:** Minimum percent decrease (Δ_min_) between initial and final bile acid concentrations that the assay can detect as statistically significant in BSH-positive microbes, depending on the number of biological replicates (N).

**N**	2	3	4	5	6	7	8
**Δ_min_ (%)**	54.4	20.6	14.3	11.6	10.0	9.0	8.2

**Table 3:** Comparison among the techniques for deconjugation activity detection in BSH-expressing microbes.

Method	Main Advantages	Main Disadvantages
Agar plate assay	Cheap and easy to carry out	Semi-quantitative; poor sensitivity; requires long incubation time (48–72 h)
TLC	Sensitive; could be automated via high-performance TLC with moderate throughput	Semi-quantitative; manual procedures required; low-throughput
HPLC	Accurately detects several bile acids; compatible with complex matrices	Low-throughput data generation
Ninhydrin assay	High-throughput (compatible with multiwell format)	Incompatible with complex matrices; sensitive to contaminations that lead to false positives
Probe-based assays	Sensitive and compatible with complex samples	Requires the synthesis of specific reagents; possible changes in deconjugation dynamics between probes and natural BAs
Method designed in this work	User-friendly and cheap; compatible with high-throughput setups	Only tested on samples from live cultures, not on in vitro reactions; matrix effects detected on calibration curves

## Data Availability

All the relevant data are contained within the article. Further inquiries can be directed to the corresponding author.

## Abbreviations

The following abbreviations are used in this manuscript:

BSH	Bile salt hydrolase
BA	Bile acid
CA	Cholic acid
TCA	Taurocholic acid
GCA	Glycocholic acid
CDCA	Chenodeoxycholic acid
TCDCA	Taurochenodeoxycholic acid
GCDCA	Glycochenodeoxycholic acid
OD	Optical density
GFP	Green fluorescent protein
ODE	Ordinary differential equation
HPLC	High-performance liquid chromatography
TLC	Thin-layer chromatography
